# Management of Postharvest Fungal Diseases in Fruits and Vegetables

**DOI:** 10.3390/jof12010029

**Published:** 2025-12-30

**Authors:** Luciana Cerioni, Viviana Andrea Rapisarda

**Affiliations:** Instituto Superior de Investigaciones Biológicas (INSIBIO), CONICET-UNT, and Instituto de Química Biológica “Dr. Bernabé Bloj”, Facultad de Bioquímica, Química y Farmacia, Universidad Nacional de Tucumán, Chacabuco 461, San Miguel de Tucumán T4000ILI, Tucumán, Argentina

## 1. Introduction

Postharvest fungal diseases pose a significant challenge across the globe. They result in the severe decay of fruits and vegetables, producing substantial economic losses throughout the harvest, transportation, storage, and marketing of fresh produce. Given the essential role played by fruits and vegetables in dietary health, as well as the increase in public concerns regarding the usage of synthetic chemical and the rise in fungicide-resistant pathogenic strains, there is growing demand for alternative and environmentally friendly postharvest management strategies.

This Special Issue, entitled “Management of Postharvest Fungal Diseases of Fruits and Vegetables”, gathers cutting-edge research addressing this challenge from multiple angles: enhancements in host defense, characterization of emerging pathogen threats, and the development of sustainable biological and nanotechnology-based control approaches.

## 2. Overview of Published Articles

A key direction in crop protection is capitalizing on intrinsic host resistance. There often remains a gap in knowledge of the complexity of plant genotype-specific responses to necrotrophic fungi. The study by Maggiolini et al. (Contribution 1) addresses this by investigating the transcriptomic responses to *Botrytis cinerea*, the causal agent of gray mold, in tolerant and susceptible grapevine (*Vitis vinifera* L.) genotypes using RNA sequencing (RNA-seq). This research offers insights into the molecular basis of grapevine resistance, highlighting metabolic plasticity as a determinant of this attribute. The tolerant genotype demonstrated enhanced modulation of metabolic processes, prioritizing secondary metabolism (e.g., phenylpropanoid biosynthesis) and stress adaptation over growth at early infection stages. Conversely, the susceptible genotype exhibited less coordinated metabolic reprogramming. The findings allowed for the identification of critical pathways, including MAPK signaling and phenylpropanoid biosynthesis, as well as of candidate genes (such as WRKY transcription factors and enzymes involved in cell wall fortification) that offer potential targets for breeding to enhance host defenses and reduce fungicide dependency.

Effective control strategies depend on the accurate monitoring and identification of prevalent pathogens as well as on the detection of possible concomitant mycotoxin contamination. The study by Dudaš et al. (Contribution 2) investigated the causal agents of apple blue mold in Serbia, confirming *Penicillium expansum* (92.9%) and *P. crustosum* (4.3%) to be the major agents. Crucially, this work reports on the identification of *P. solitum* and *P. chrysogenum* as causal agents for the first time in Serbia, revealing a broader diversity of pathogens associated with the disease. Furthermore, the study addressed the public health threat posed by mycotoxins, detecting the presence of the *msas* gene and patulin production exclusively in all *P. expansum* isolates. Moreover, the research conducted by Dutta and colleagues (Contribution 3) confirmed the active prevalence of *Fusarium falciforme* and *F. acutatum* in causing Fusarium basal rot (FBR) in onion in Maharashtra, India. Historically, FBR in this region was majorly caused by *Fusarium oxysporum* f. sp. *cepae*. The identification and confirmation of these specific species as independent causal agents in the region represent the first report from Maharashtra and underscore the ongoing challenge of the changing pathogen populations.

Addressing the limitations of synthetic fungicides, particularly the emergence of resistance in pathogens, requires the development of sustainable and innovative “green” technologies. This Special Issue features two promising alternative approaches: nanoparticle technology and biocontrol agents (BCAs). Baigorria et al. (Contribution 4) assessed the antifungal action of metallic nanoparticles (NPs) against key postharvest lemon pathogens, including fungicide-sensitive and fungicide-resistant strains of *P. digitatum* and *P. italicum*, and *Geotrichum citri-aurantii*. Their study found that silver NPs (Ag-NPs) demonstrated higher efficacy (MFC of 10 µg mL^−1^) compared to copper oxide NPs (CuO-NPs) (MFC of 1000 µg mL^−1^) against conidia. The mechanism of action of NPs involves disruptions to conidial membrane permeability and severe intracellular disorganization. Ag-NPs were effective even against the fungicide-resistant isolates and reduced mold incidence on inoculated lemons, suggesting their potential as a new alternative control strategy. In addition, Gomomo and coauthors (Contribution 5) explored the efficacy of specific non-Saccharomyces yeasts (*Suhomyces pyralidae*, *Pichia kluyveri*, and *Aureobasidium melanogenum*) as sustainable biocontrol solutions against *B. cinerea* on apples and strawberries. The results confirmed their potential, with inhibition rates varying by yeast strain and fruit type. Mechanisms included the secretion of extracellular lytic enzymes (e.g., glucanase, protease, chitinase), prominently demonstrated by *A. melanogenum*, and the production of volatile organic compounds (VOCs). The antagonistic mechanism of *P. kluyveri* was linked primarily to VOCs, such as isobutanol and isoamyl acetate, which contributed to fungal inhibition. Notably, *S. pyralidae* and *P. kluyveri* achieved 100% inhibition against certain *B. cinerea* strains on strawberries, matching the efficacy of the commercial fungicide Captan.

## 3. Outlook and Future Directions

The integration of research across the domains of host molecular biology, pathogen dynamics, and novel control technologies is critical for the development of resilient and sustainable agricultural systems. The studies presented in this Special Issue effectively address several key challenges, while also opening up new avenues for investigation.

In the future, functional validation of the candidate genes identified in resistant cultivars (such as in grapevine) would be essential for their effective utilization in marker-assisted selection or genetic engineering programs. Furthermore, while biological and nano-based controls demonstrate high promise, future research must focus on the safety assessment, formulation, and stability of agents such as metallic NPs and VOCs/BCAs for large-scale commercial application. This should include assessing their effectiveness and stability under varying environmental conditions and evaluating their use in combined strategies featuring conventional chemical treatments to reduce dosages and mitigate resistance development. Finally, the continuous monitoring and diagnostics of evolving pathogen populations, such as the changing *Fusarium* species complex in onion, and the assessment of mycotoxin accumulation across different *Penicillium* species remain paramount to maintaining food safety and adapting management protocols in specific geographical areas.

In conclusion, this Special Issue provides a dynamic vision of the multidisciplinary efforts required to secure crop health and food security in the face of persistent and emerging fungal threats. These novel findings lay the groundwork for reducing reliance on chemical treatments to reduce postharvest losses of fruits and vegetables and promote sustainable agriculture globally.

The Editors extend their sincere gratitude to all contributing authors, reviewers, and editorial staff, whose valuable efforts were crucial in the successful publication of this Special Issue.

